# Immunosuppressive phenolic compounds from *Hydnora abyssinica* A. Braun

**DOI:** 10.1186/s12906-015-0931-x

**Published:** 2015-11-09

**Authors:** Waleed S. Koko, Mohamed A. Mesaik, Rosa Ranjitt, Mohamed Galal, Muhammad I. Choudhary

**Affiliations:** College of Science and Arts in Ar Rass, University of Qassim, Qassim, KSA Saudi Arabia; Medicinal and Aromatic Plants Research Institute, National Center for Research, Khartoum, Sudan; Dr. Panjwani Center for Molecular Medicine and Drug Research, Khartoum, Sudan; Drug and Herbal Research Centre, Faculty of Pharmacy, Universiti Kebangsaan Malaysia, Jalan Raja Muda Abdul Aziz, 50300 Kuala Lumpur, Malaysia; H. E. J. Research Institute of Chemistry, International Center for Chemical and Biological Sciences, University of Karachi, Karachi, 75270 Pakistan

**Keywords:** *Hydnora abyssinica*, Immunosuppression, Chemiluminescence assay, Oxidative burst

## Abstract

**Background:**

*Hydnora abyssinica* (HA) A. Braun is an endemic Sudanese medicinal plant traditionally used as anti-inflammatory and against many infectious diseases. However, it proved to be very rich in phenols and tannins, so the present study was undertaken to investigate the immunomodulatory potential of the whole plant ethanolic extract and its isolated compounds.

**Methods:**

Lymphocyte proliferation, chemiluminescence and superoxide reduction assays were used for immunomodulatory evaluation. While, MTT (3–(4, 5-dimethylthazol-2-yl)-2, 5-diphenyl tetrazonium bromide) test was performed on 3 T3 cell line clone in order to evaluate the cytoxicity effect of the extracts and isolated compounds of phenolic derivatives which were carried out by chromotographic techniques.

**Results:**

Catechin, (1), tyrosol (2) and benzoic acid, 3, 4, dihydroxy-, ethyl ester (3) compounds were isolated from HA ethanolic extract which revealed potent immunosuppressive activity against reactive oxygen species from both polymorph nuclear cells (PMNs) (45–90 % inhibition) and mononuclear cells (MNCs) (30 -65 % inhibition), T lymphocyte proliferation assay (70–93 % inhibition) as well as potent inhibitory effect against superoxide production (42–71 % inhibition) at concentrations of 6.25–100 μg/mL. Catechin (**1**) was found the most potent immunosuppressive agent among all constituents examined.

**Conclusion:**

These results can support the traditional uses of *H. abyssinica* extracts as anti-inflammatory and immunosuppressive and further investigations of the mode of action and other pharmacological studies are highly desirable.

## Background

Immunosuppressive drugs are commonly prescribed to prevent the rejection of transplanted organs, or to treat chronic inflammatory diseases, such as ulcerative colitis, neutropenia, Crohn’s disease, rheumatoid arthritis and connective tissue disorders [1–3].

From centuries people has been using the herbal medicines for the treatment of various diseases [4, 5]. Up today, ethnomedicine still plays the axial role in the search and development of new drugs [6]. Similarly for many developing countries, herbal drugs have a major importance based on indigenous knowledge, cultural acceptability and affordability [7]. Indeed, it is estimated that about 90 % of Sudanese population depend mainly on herbal medicines for the primary treatment of various types of diseases, ranging from simple disorders to more chronic pathologies such as cancers [8].

*H. abyssinica* belongs to the family Hydronaceae (Leafless and rootless parasite). The plant is locally known as “Tartous”, and distributed in central Sudan. In Sudanese folk medicine, the whole plant aqueous extract is used for the treatment of swellings, tonsillitis and dysentery [9]. The plant aqueous extract had shown considerable molluscicidal activities against *Bulinus truncatus* and *Biomphalaria pfeifferi* [10]. Recently preliminary phytochemical analysis of *H. abyssinica* has shown the latter to be very rich in phenols and tannins [11].

In spite of its ethnobotanical uses, there is a paucity of pharmacological and chemical data on Sudanese tartous. To this effect, the present study was therefore undertaken to investigate the immunomodulatory activity of the whole plant ethanolic extract and its isolated secondary compounds through lymphocyte proliferation, chemilumenscence and superoxide reduction assays. MTT cytotoxicity against 3 T3 cell line carried out for plant ethanolic extracts and the isolated compounds.

## Methods

### Plant material

The selected plant species was collected from its natural habitat in the central Sudan around *Acacia* sp. Voucher specimens was identified by Dr. Wai’l S. Abdalla and Mr. Haidar Elsidig of Herbarium of Medicinal and Aromatic Plants Research Institute (MAPRI), Khartoum, Sudan, where the specimen was also deposited (MO22–07).

### Preparation of the crude extract

Hundred grams of whole plant material was air-dried under the shed, grounded, and extracted by triple soaking in 80 % ethanol at room temperature for 3 days. Extracts were obtained by removing the organic solvent under reduced pressures by using rotary evaporator.

### Isolation of secondary metabolites (constituents)

#### Spectroscopic techniques

UV Spectra were recorded in methanol or chloroform on a Hitachi UV 3200 spectrophotometer. IR Spectra were recorded in CHCl_3_ or KBr on a Jasco A-302 IR spectrophotometer. The EI MS spectra were recorded on a double focusing mass spectrometer (Varian MAT 311A). HREI MS measurements were performed on a Jeol HX 110 mass spectrometer. The ^1^H-NMR spectra were recorded on Bruker AC-300 and AM-400 MHz instruments, while ^13^C-NMR spectra were recorded at 100 and 125 MHz. Multiplicities of the carbon signals were determined by DEPT 90° and 135° experiments. ^1^H-NMR and ^13^C-NMR chemical shifts were reported in δ (ppm) and coupling constants (*J*) were measured in Hz.

### Chromatographic techniques

Column chromatography was performed on A silica gel 60 (70–230 and 240–300 mesh sizes, E. Merck), Diaion HP-20 Resin and Sephadex LH-20 were used. Final purification of some compounds was achieved by preparative recycling HPLC (model LC-908) (JAI) and the columns used were L-80 or H-80 (YMC, Co. Ltd) at appropriate flow rates (4 mL/min) using equivalent combinations of MeOH and H_2_O. Pre-coated silica gel TLC plates (E. Merck, F_254_) were used for evaluating the compounds. TLC plates were viewed under ultraviolet light at 254 nm for fluorescence quenching spots and at 366 nm for fluorescent spots. Ceric-sulphate in 10 % H_2_SO_4_ spraying reagent was used for staining the compounds on TLC.

### Isolation and purification of compounds

**Fig. 1:**
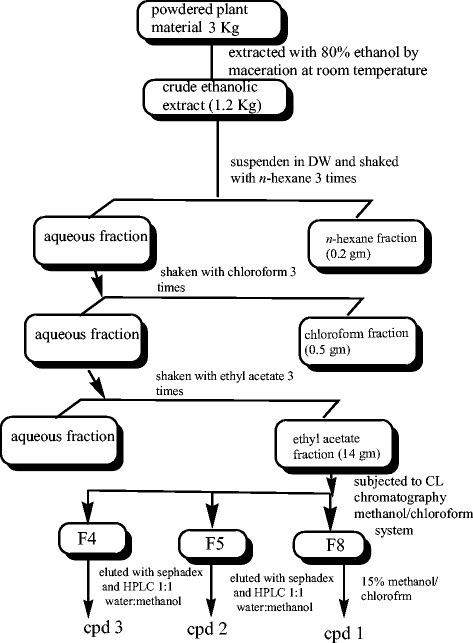
Isolation of secondary metabolites from *H. abyssinica*

Dried powder of the whole plant (3 Kg) was extracted exhaustively with 80 % ethanol at room temperature. The filtrate was evaporated under vacuum reduced pressure to yield (1.2 Kg). The residue was suspended in distilled water (partially soluble) then partitioned in different solvents on the basis of increasing polarity (solvent solvent system of fractionation or liquid-liquid extraction) using big size separating funnel (5 L) to get *n*-hexane (0.2 g), chloroform (0.5 g), ethyl acetate (14 g) and *n*-butanol (60 g), (Fig. 1)

The crude ethyl acetate fraction (14 g) was subjected to column chromatography eluted with 50 % chloroform/*n*-hexane with gradient increase of chloroform up to 100 and methanol up to 100 %. Ten fractions were obtained. On repeated flash column chromatography using chloroform/methanol system for fraction (8), compound 1 was obtained by elution column with 15 % methanol/chloroform.

Fractions 4 and 5 were loaded on reverse phase Sephadex column LH-20 starting by high polarity 100 % distilled water and followed by a gradient decrease with methanol until 100 % methanol, from each fraction 3 sub fractions were obtained, the sub fraction (2) of each were subjected to recycling preparative high performance liquid chromatography (HPLC), using mobile phase of water-methanol (1:1) on L-80 ODS column (reverse phase). The purification of compounds 2 and 3 was conducted by using repeated recycling HPLC.

### Bioassay techniques

#### Isolation of Human Polymorphoneutrophils (PMNs) and Mononuclear Cells (MNCs)

Heparinized blood was obtained by vein puncture aseptically from healthy volunteers (25–38 years age). The consent was obtained from the healthy donors for the use of their blood in this study. The buffy coat containing PMNs and MNCs was collected by dextran sedimentation and the cells were isolated after the Lymphocyte Separation Medium (LSM) density gradient centrifugation. MNCs were separated gently by collecting them from the plasma- ficoll interface using pasteur pipette, while PMNs were collected from the tube base. Cells were washed twice and suspended in Hank’s Balance Salt Solution [Ca and Mg free] (HBSS^−−^), pH 7.4. Neutrophils were purified from RBCs contamination using hypotonic solution. Cells were adjusted to their required concentration using Hank’s Balance Salt Solution containing Ca and Mg (HBSS^++^). The study was approved by the local Ethics Committee (MAPRI Ethics Committee, National Centre for Research, Khartoum, Sudan).

### Chemiluminescence assay

Luminol or lucigenin–enhanced chemiluminescence assay were performed as described by Helfand et al. [12] and Haklar et al. [13]. Briefly, 25 μL of PMNCs (1 × 10^6^) and MNCs (5 × 10^6^) cells were incubated with 25 μL of serially diluted plant extract with concentration ranges between 6.25 to 100 μg/mL. while concentrations of 3.13 to 50 μg/mL were used for pure compounds. Predinsalone was used as reference drug (control positive) with concentration of 10 μg/mL. Control wells (control negative) received HBSS^++^ and cells but no extract. Tests were performed in white 96 wells plates in triplicate for each concentration and controls, which were incubated at 37 ° C for 30 min in the thermostated chamber of the luminometer. Opsonized zymosan-A or PMA 25 μL, followed by 25 μL luminol (7 × 10^−5^M) or lucigenin (0.5 mM) along with HBSS^++^ was added to each well to obtain a 200 μL volume/well. The luminometer results were monitored as chemiluminescence RLU (relative light unit), with peak and total integral values set with repeated scans at 30 s intervals and one second points measuring time.

### T-cell proliferation assay

Cell proliferation was evaluated by standard thymidine incorporation assay following a method reported by Nielsen et al. [14]. Briefly, cells were obtained from peripheral blood of healthy individuals (the consent was obtained from the healthy donors for the use of their blood in this study which was approved by the local Ethics Committee (MAPRI Ethics Committee, National Centre for Research, Khartoum, Sudan). Then cultured at a concentration of 5 × 10^5^/mL in a 96-well round bottom tissue culture plates (Nalge Nunc. Inter. USA). Preliminary experiments were conducted to determine the optimum concentration of PHA on T-cell proliferation. PHA concentration 5 μg/mL was found to be optimum and used in our experiment. Cells were stimulated with 5 μg/mL of PHA (Sigma-Aldrich Co., USA). Various concentrations of plant extracts were added to obtain final concentrations of 6.25, 12.5, 25, 50 and 100 μg/mL, each in triplicate. In another set of experiment cells were incubated with half concentrations of compounds and predinsalone was used as reference drug (control positive) with concentration of 10 μg/mL. After 24 h, cells were washed twice and then stimulated with 5 μg/mL PHA. The plates were incubated for 72 h at 37 °C in 5 % CO_2_ incubator. After 72 h, cultures were pulsed with (0.5 μCi/well) [H^3^] tritiated thymidine (Amersham Pharmacia Biotech, USA), and further incubated for 18 h. Cells were harvested onto a glass fiber filter (Cambridge Technology, USA) using cell harvester (SKATRON A.S. Flow Lab., Norway). The tritiated thymidine incorporated into the cells was measured by a liquid scintillation counter (LS 6500, Beckman Coulter, USA). Results were expressed as mean count per minute (CPM). The inhibitory activity of T lymphocyte proliferation was calculated using the following formula:

$$ Inhibitory\  activity\ \left(\%\right) = 100\ x\frac{Control\  group\ (CPM)- Experiment\  group\ (CPM)}{Control\  group\ (CPM)} $$

### Superoxide anion radical scavenging activity

The experiment was carried out in 96 microtiter ELISA plates with complete volume of 200 μL/ well. 10 μL of tested samples were dissolved in DMSO (final concentration of 1 mM for pure compounds and the above mentioned 5 concentrations for the plant extract) in this experiment. For the generation of the superoxide radicals 90 μL of phosphate buffer (0.1 M, pH 7.5) were added, followed by the addition of 40 μL (80 mM) of NBT (nitroblue tetrazolium) solution and 40 μL (280 mM) NADH (nicotinamide adenine dinucleotide hydrogen reduced form) and the reaction was started by adding 20 μL (8 mM) of PMS (phenazine methosulphate) solution to the mixture. The reaction mixture was incubated at 25 °C for 5 min and the absorbance at 560 nm was measured against blank samples. Propyl gallate was used as a positive control and DMSO as negative control. Decreased absorbance of the reaction mixture indicated increased superoxide anion scavenging activity. The inhibition percentage of superoxide anion generation was calculated by using the following formula:

$$ \% Inhibition = 100\ x\frac{Control\  group\ (OD)- Experiment\  group\ (OD)}{Control\  group\ (OD)} $$

### MTT cytotoxicity test

Serial dilutions of compound was prepared in a 96-well flat bottomed plate (Nalge Nunc, Inter. USA). The outer wells of the plate were filled with 250 μL of in-complete culture medium, except the last row 6 middle wells (B - G), which were used for the negative control receiving 50 μL of culture medium and 2 μL of sterile 0.5 % Triton X.

To the rest of the plate, 50 μL/wells (CCM) were added and 30 μL more were added to second column wells (B – G) that were used as first compounds dilution wells. To the first dilution wells in the row, 20 μg of compound suspension were added to the 80 μL. Compounds were then serially diluted by two-fold dilution from well B3 till B11 by transferring 50 μL to the next well after proper mixing. From the last dilution wells (B-11), 50 μL were discarded. Each compound was tested in triplicate. Cell suspension in a complete culture medium containing 2.5 × 10^5^/mLwas properly mixed, and 150 μL of it were transferred into each well of the plate. The plate was covered and placed in 5 % CO_2_ incubator at 37 °C for three-five days (72–120 h). On the third/fifth day, the supernatant was removed from each well without detaching cells. MTT stock (5 mg/mL) was prepared earlier in 100 mL PBS. MTT suspension was vortexed and kept on magnetic stirrer until all MTT dissolved. The clear suspension was filter sterilized with 0.2 μm Millipore filter and stored at 4 °C or –20 until use. MTT was diluted in a culture medium (1:3.5) and brought to room temperature. To each well of the 96-well plates, 50 μL of diluted MTT were added. The plate was incubated further at 37 ° C for 2 to 3 h in CO_2_ incubator. MTT was removed carefully without detaching cells, and 200 μL of DMSO were added to each well. The plate was agitated at room temperature for 15 min then read at 540 nm using microplate reader.

### Statistical analysis

All data are presented as means ± standard deviation of the mean. Statistical analysis for all the assays results were done using student’s *t*-test. Significance was attributed to probability values P ≤ 0.05 or P ≤ 0.001 in some cases.

## Results

### Isolated compounds

#### Catechin (1)

It is a well known polyphenolic secondary metabolite, isolated for the first time from *H. abyssinica*, ethyl acetate (bulk amount 2 g) fraction by using 25 mm diameter column chromatography (silica gel) in chloroform methanol system, purified from sub fraction column 8 in 50 % ethyl acetate/chloroform.

Physical status: White crystals

Yield: 6.7 X 10^−2^%

R_f_: 0.7 (15 % Chloroform/methanol)

Melting point: 241–243 °C.

[α]_D_^25^:−45 °(c = 0.1, CH_3_OH)

IR (KBr) ν_max_ cm^−1^: 3420 (OH), 1646 (C = C).

^1^H NMR (CD_3_OD, 300 MHz, δ): 6.83 (1H, d *J* = 1.7 Hz, H-2′), 6.75 (1H, d *J* = 8.1 Hz, H-5′), 6.72 (1H, d *J* = 1.8 Hz, H-6′), 5.9 (1H, d *J* = 2.2 Hz, H-8), 5.8 (1H, d *J* = 2.2 Hz, H-6), 4.55 (1H, d *J* = 7.5 Hz, H-2), 3.98 (1H, dt, *J* = 7.6 Hz, *J* = 5.3 Hz H-3), 2.87 (2H, dd, *J* = 5.5,10.7 Hz H-4).

^13^C NMR (CD_3_OD, 100 MHz, δ): 157.5 (C-7), 157.3 (C-5), 156.0 (C-9), 146.2 (C-3′ and 4′), 132.2 (C-1′), 120.0 (C-6′), 116.3 (C-2′), 100.8 (C-10), 96.3 (C-6), 95.5 (C-8), 82.2 (C-2), 68.8 (C-3), 28.5 (C-4)

HREI MS: *m*/*z* 290.272 (calcd 290.0790 for C_15_H_14_O_6_)

EI MS (rel.int. %): *m/z* 290 (16), 167 (3), 152 (44), 139 (100), 124 (17), 110 (9), 77 (14), 69 (26).

### 2-(4 hydroxyphenyl) ethanol (Tyrosol) (2)

It is a simple phenol derivative compound isolated from *H. abyssinica* ethyl acetate fraction by using recycling P-HPLC (1:1) water: methanol L-80 ODS reversed phase column.

Physical status: Colorless needles

Yield: 2.7 X 10^−4^%

R_f_: 4.7 (10 % chloroform/methanol)

Melting point: 85–86 °C.

IR (CH_3_OH) ν_max_ cm^−1^: 3380, 3140 (OH), 1590 (C = C)

^1^H NMR (CD_3_OD, 400 MHz, δ): 7.0 (1H, d, *J* = 2.1 Hz, H-2 and 6), 6.7 (1H, m, H-3 and H-5), 3.6 (2H, t, *J* = 7.2 Hz, H-8), 2.7 (2H t, *J* = 7.2 Hz, H-7).

^13^C NMR (CD_3_OD, 100 MHz, δ): 159.2 (C-4), 131.3 (C-1), 130.8 (C-3 and 5), 116.1 (C-2 and C-6), 64.0 (C-8), 40.0 (C-7).

HREI MS: *m/z* 138.66 (calcd 138.0681 for C_8_H_10_O_2_)

EI MS (rel.int. %): *m/z* 138 (39), 121 (32), 107 (100), 93 (8), 77 (44).

### Benzoic acid, 3, 4, dihydroxy- ethyl ester (3)

It is benzoic acid and phenol derivative, isolated from *H. abyssinica* ethyl acetate fraction by using recycling P-HPLC (1:1) water: methanol L-80 ODS reversed phase column.

Physical status: Colorless crystal

Yield: 3.4 X 10^−4^%

R_f_: 6.5 (10 % chloroform/methanol)

Melting point: 130–135 °C.

IR (KBr) ν_max_ cm^−1^: 3660 (OH), 1765 (C = O), 1587 (C = C), 1250 (C-O)

^1^HNMR (CD_3_OD, 400 MHz, δ): 7.4 (1H, dd, *J* = 8.8 Hz, *J* = 1.0 Hz, H-5), 6.7 (1H, d, *J* = 8.0 Hz, H-2), 4.3 (2H, m, H-8), 1.3 (3H, m, Me-9).

^13^C NMR (CD_3_OD, 100 MHz, δ): 168.4 (C-7), 151.8 (C-1), 146.2 (C-6), 123.5 (C-3), 122.8 (C-4), 117.4 (C-2),115.8 (C-5), 61.6 (C-8), 14.7 (C-9).

HREI MS: *m/z* 182.176 (calcd 182.0579 for C_8_H_10_O_2_)

EI MS (rel.int. %): *m/z* 182 (35), 153 (17), 137 (100), 109 (45), 77 (52).

### Chemiluminescence assay

**Fig. 2:**
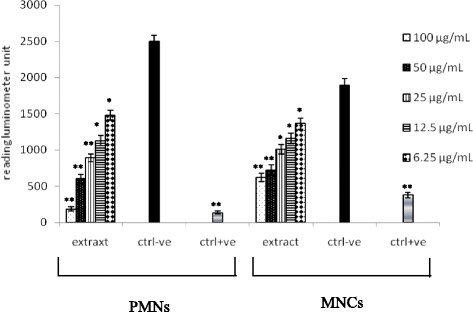
Inhibitory properties *H. abyssinica* ethanolic extracts for PMNs and MNCs activated by SOZ (luminol base)

**Fig. 3:**
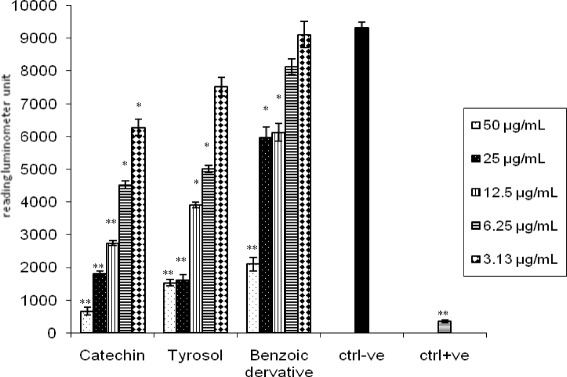
Inhibitory properties of isolated phenolic compounds for PMNs activated by SOZ (luminol based)

The preliminary screening results of *H. abyssinica* ethanolic extract on both isolated immune cells types,PMNs and MNCs was carried out after activation of cells using serum opsonized zymosan (SOZ) and followed by luminol as indicator. The dose dependent inhibitory properties for phagocytosis was reported for all tested concentrations (6.25 to 100 μg/mL) against both PMNs (45–90 % inhibition) and MNCs (30–65 % inhibition) Fig. 2. The inhibitory effects of the isolated compounds on PMNs activated by SOZ was found 92, 79 and 95 % with higher concentration {50 μg/mL} and 30, 25 and 5 % with the lower concentration {3.15 μg/mL} for the compounds catechin (0.17–0.01 mM), tyrosol (0.36–0.02 mM) and benzoic acid derivative (0.27–0.02 mM), respectively Fig. 3.

### T-cell proliferation assay

**Fig. 4:**
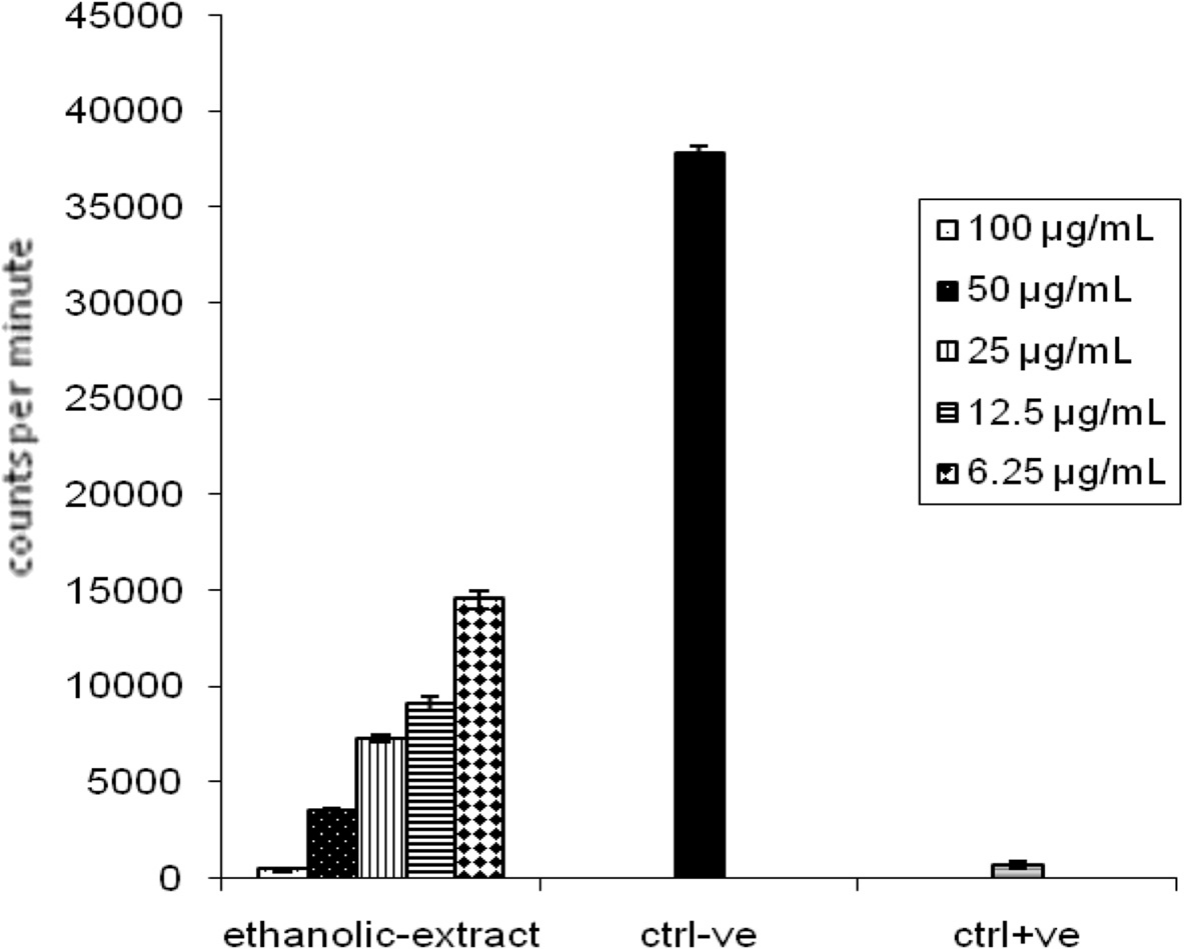
Inhibitory properties of *H. abyssinica* ethanolic extract for T lymphocyte proliferation the cells were stimulated with PHA

**Fig. 5:**
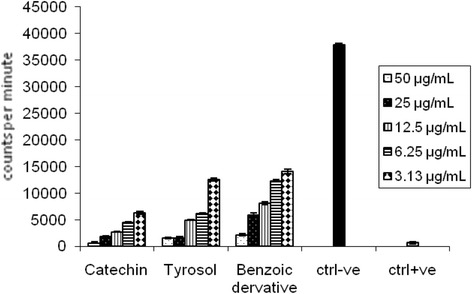
Inhibitory properties of isolated phenolic compounds for T lymphocyte proliferation the cells were stimulated with PHA

The effects of *H. abyssinica* extract and isolated compounds on ^3^H-thymidine incorporation into PHA (phytohaemagglutinin)-stimulated peripheral blood mononuclear cells are presented in Figs. 4 and 5. Inhibitory % of T-cell proliferation for *H. abyssinica* ethanolic extract was found in the range 67-97 % for the tested concentrations 6.25–100 μg/mL. The inhibitory % of isolated compounds was found in the range of 83–98, 71–95 and 66–94 % for the tested concentrations (3.15–50 μg/mL) of catechin, tyrosol and benzoic acid derivative, respectively.

### Superoxide anion radical scavenging activity

**Fig. 6:**
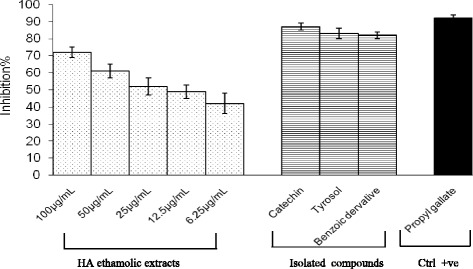
Inhibitory properties of *H. abyssinica* ethanolic extract and isolated compounds for superoxide production

The plant ethanolic extract was screened for its superoxide radicals scavenging by using 5 concentrations (6.25 – 100 μg/mL). These concentrations had shown inhibitory activity for superoxide production ranging from 42 to 72 % inhibition (Fig. 6). While the isolated compounds revealed 87, 83 and 82 % inhibition for catechin, tyrosol and benzoic acid derivative respectively (Fig. 6).

### MTT cytotoxicity test

Neither the plant ethanolic extract nor isolated compounds revealed cytotoxic activity against 3 T3 cell line (Table 1).

**Table 1:** MTT reduction cytotoxic assay for evaluation of *H. abyssinica* and its isolated compounds for their inhibition % against 3T3 cell line

Conc. in μg/mL	Compound (1)	Compound (2)	Compound (3)	Ethanolic extract
50	17.8 + 3.3	17.2 + 3.7	15.8 + 5.1	12.4 + 4.2
25	14.4 + 4.1	14.2 + 2.8	12.5 + 4.3	11.5 + 3.5
12.5	10.8 + 3.6	12.1 + 3.1	11.4 + 4.1	10.2 + 2.7
6.25	8.1 + 2.8	4.0 + 1.3	11.9 + 3.8	8.7 + 2.3
3.13	1.4 + 0.3	3.4 + 0.4	4.7 + 1.1	-
Triton X	66.7 + 11.6			

This table indicates the activity of HA ethanolic extract and isolated compounds on the viability of 3T3 cell line after 72 h incubation with and without five concentrations (6.25–100 μg/mL) of the plant extract and (3.13–50 μg/mL) for isolated compounds. Triton X 0.5 % was used as control positive. Cells viability was evaluated by MTT reduction to the blue colored formazan in living cells which was detected by measuring absorbance at 540 nm against blank samples. The % inhibition was calculated as: % *Inhibition* = [(*AControl* − *ASample*)/*AControl*] × 100

Where AControl is the absorbance of the negative control and ASample the absorbance of tested samples or standard. Readings are presented in mean ± SD of three repeats

## Discussion

Since immemorial time plants have been used as an alternative therapy and has stood the test of time. Indeed, many medicinal plants are traditionally used to control unregulated immune stimulation in the cases of anaphylactic shock [15], inflammatory response, asthma, etc. [16, 17].

During the present investigation, the ethanolic extract of *H. abyssinica* whole plant showed a significant suppression towards the immune response activation which was assayed by oxidative burst chemiluminescence for both polymorph nuclear cells (PMNs) (45–90 % inhibition) and mononuclear cells (MNCs) (30–65 % inhibition) activated with SOZ. However, in the second assay, T- cell proliferation activity was inhibited significantly (67–97 % inhibition) with all tested concentrations (6.25–100 μg/mL). Also these concentrations were found to be of a significant inhibition for superoxide free radicals (42 to 72 % inhibition). Superoxide radicals scavenging assay was conducted to evaluate immunsupressive activity due the fact that all reactive oxygen species play a major role in the long-term complications of immune-related diseases [18, 19]. However, in the present work, SOZ was used for the stimulation phagocytic cells for superoxide and others reactive oxygen species production. Luminol is characterized by its ability to enter the cell and reacts with intracellular reactive oxygen species (ROS) [20, 21].

The above study on *H. abyssinica* ethanolic extract encourage further investigation on the plant for the isolation of secondary metabolites, responsible of these activities. Interestingly, these three well known phenolic compounds were isolated for the first time from *H. abyssinica*. All of these compounds showed suppression of oxidative burst (79–95 % inhibition) for the highest concentration 50 μg/mL and 5–30 % inhibition for the lowest concentration (3.2 μg/mL). All of them revealed potent inhibition for T-cell proliferation (94–98 % inhibition) for the highest concentration (50 μg/mL) and (66–83 % inhibition) for the lowest concentration (3.2 μg/mL). Also they showed inhibition for superoxide production (82–87 %). Results reported from the present study are consistent and in line with the previous observation of Wang et al. [22]. They indicated that aromatic compounds are extremely active in suppressing immune responses in both in-vitro and in-vivo.

Catechin (1) was found the most potent immunosuppressive, among all the compounds tested. Previous studies indicated that catechin is the major polyphenol component of green tea, which has attracted a considerable attention during the last years. It is a powerful antioxidant and the significant activities against all diseases are found associated with ROS productions [23, 24]. Neither *H. abyssinica* extract nor isolated compounds possesed cytotoxicity activity against 3 T3 cell line. Although they were revealed dose dependant cytotoxic effect but the maximum examined concentration was found of less than 20 % and the IC_50_ going to be more than 10000 μg/mL, which is 200 times the higher concentrations examined.

## Conclusion

In our conclusion we can support the traditional uses of *H. abyssinica* extracts as anti-inflammatory and immunosuppressive, mainly due to the dominant quantities of catechin (>0.07 %) and other phenolic compounds. Further investigations of the mode of action and other pharmacological studies such like the level of various cytokines in the peripheral blood that is possible to suggest for understand of immune response activity also more experimental analysis for the characterization of immune response activity by the catechin are highly desirable.
